# Graph Multi-Scale Permutation Entropy for Bearing Fault Diagnosis

**DOI:** 10.3390/s24010056

**Published:** 2023-12-21

**Authors:** Qingwen Fan, Yuqi Liu, Jingyuan Yang, Dingcheng Zhang

**Affiliations:** 1 School of Mechanical Engineering, Sichuan University, Chengdu 610017, China; fanqingwen@scu.edu.cn (Q.F.); yuqi_liu@stu.scu.edu.cn (Y.L.); 2 School of Engineering, University of Birmingham, Birmingham B152TT, UK; jxy840@student.bham.ac.uk

**Keywords:** roller bearing, fault diagnosis, multi-scale permutation entropy, graph entropy

## Abstract

Bearing faults are one kind of primary failure in rotatory machines. To avoid economic loss and casualties, it is important to diagnose bearing faults accurately. Vibration-based monitoring technology is widely used to detect bearing faults. Graph signal processing methods promising for the extraction of the fault features of bearings. In this work, graph multi-scale permutation entropy (MPE_G_) is proposed for bearing fault diagnosis. In the proposed method, the vibration signal is first transformed into a visibility graph. Secondly, a graph coarsening method is used to generate coarse graphs with different reduced sizes. Thirdly, the graph’s permutation entropy is calculated to obtain bearing fault features. Finally, a support vector machine (SVM) is applied for fault feature classification. To verify the effectiveness of the proposed method, open-source and laboratory data are used to compare conventional entropies and other graph entropies. Experimental results show that the proposed method has higher accuracy and better robustness and de-noising ability.

## 1. Introduction

Bearings play an important role in many machines, such as aero-engines, rail vehicle bogies, wind-driven generators, etc., for transmitting speed and carrying radical load. The working conditions of bearings are harsh, i.e., variable load and speed, which results in bearing failures occurring easily. Bearing faults may lead to shutdown of the whole machine, causing economic loss and even casualties. To avoid tragedies, many condition monitoring technologies, such as vibration-based detection, acoustic emission-based detection, etc., have been used to detect early faults in bearings. Vibration signals have rich physical features including fault information and hence vibration-based monitoring technology has been widely used in bearing fault diagnosis.

The main bearing fault diagnosis methods which use vibration signals can be divided into two types, i.e., signal processing-based and machine learning-based methods [1,2]. For signal processing-based methods, vibration signals are normally decomposed for de-noising or feature extraction and then the analyzed signal is processed using time-series or spectrum methods. Finally, bearing fault diagnosis can be achieved by calculating indices or observing fault features in the obtained spectrum. Some signal processing methods, such as variational mode decomposition [3], wavelet transform [4], sparse representation [5], and their variants, have been successfully applied for bearing fault diagnosis. Those methods have achieved good performance in many cases. However, the results of signal processing-based methods are highly reliant on expert experience.

For machine learning-based fault diagnosis methods, statistical models are built and trained using signal or feature samples with different health conditions. Intelligent fault diagnosis models can be completed after evaluation by testing samples. Deep learning is one kind of statistical model which has been widely applied to bearing fault diagnosis [6]. Deep learning is an “end-to-end” model which can be trained by feeding signal samples directly into the model. Many deep learning models, for example, convolution neural networks [7], stacked autoencoders [8], deep belief networks [9], and recurrent neural networks [10], have achieved good performance when using large volumes of data. However, there are few samples which include faulty conditions and many samples which demonstrate healthy conditions in real operating conditions. The problem of imbalanced sample data results in overfitting and low accuracy in deep learning models.

The bearing fault diagnosis methods which use conventional statistical models, such as k-nearest neighbor [11], Bayesian classifier [12], and support vector machines (SVM) [13], mitigate these challenges by relying on well-defined mathematical principles and feature spaces that are less susceptible to the effects of imbalanced data [2]. Unlike deep learning methods, these statistical models only require a small number of features extracted from signal samples for fault diagnosis. Feature extraction maps original signals onto the statistical parameters which convey information about the machine’s status. The selection of statistical parameters directly influences the results of fault diagnosis methods using statistical models. Statistical parameters can be divided into two different classes: physical parameters and virtual parameters [14]. Physical parameters are relevant to physics-based information about health status and are extracted from signals using signal processing methods or statistical methods, for example, root mean square, peak value, kurtosis, and entropy. Virtual parameters are obtained by fusing multiple physical parameters or multi-sensor signals by using reduction techniques such as principal component analysis, self-organizing maps, and hidden Markov models. Hence, virtual parameters lose physical meaning and just represent a virtual index related to health status.

Entropy, as one kind of physical parameter, is the measure of the disorder of a signal, and has been successfully applied in fault diagnosis as approximate entropy, sample entropy, fuzzy entropy, etc. [15]. However, the above entropies have limitations. For example, approximate entropy is restricted by the length of the signals; sample entropy heavily depends on the similarity tolerance threshold; and the boundary width of the fuzzy function in fuzzy entropy is difficult to define [15]. Based on spectrum graph theory, graph entropies can be used for analyzing data with topology structures which have rich structural information compared with entropies in Euclidean space [16]. Graph entropies have been used in different research areas, for instance, brain science [17] and web networks [18]. However, there are few research works about fault diagnosis using graph entropies.

Inspired by multi-scale permutation entropy and graph permutation entropy (PE_G_) [19], graph multi-scale permutation entropy (MPE_G_) is proposed for the fault diagnosis of bearings in this work. In the proposed method, vibration signals are first transformed into visible graphs. Secondly, the spectral graph coarsening method is introduced to obtain coarse-grained graphs with different scales. Thirdly, the PE_G_ of each coarse-grained graph is calculated and, hence, MPE_G_ can be obtained. Finally, a support vector machine (SVM) is used for classing MPE_G_ and diagnosing bearing faults. The main contributions of this work are (1) graph multi-scale permutation entropy is proposed by combining the graph coarsening method and the graph permutation entropy; (2) graph entropy is introduced for the first time for fault diagnosis.

## 2. Methodology

### 2.1. Horizontal Visibility Graphs

An undirected weighted graph can be expressed as $G=\left\{V,E,W\right\}$, where $V=\left(v_{1},v_{2},v_{3},\ldots ,v_{N},|V|=N\right)$ is a set of vertices; $E=\left(e_{1},e_{2},e_{3},\ldots ,e_{M},|E|=M\right)$ is a set of edges connecting vertices; and $W={\left(w_{ij}\right)}_{N\times N}$ is the weighted adjacency matrix, where $w_{ij}$ is the weight of the edge connecting $v_{i}$ and $v_{j}$. The Laplacian matrix of graph G is given by L=D−W, where $D=\left[d_{1},d_{2},d_{3},\ldots ,d_{N}\right]$ is the degree diagonal matrix and where $d_{i}=\sum w_{ij}$. L is a real symmetric matrix with $L=U\Lambda U^{T}$, in which $U=[u_{1},u_{2},\ldots ,u_{N}]$ refers to orthogonal eigenvectors and Λ is the diagonal matrix of a set of non-negative real eigenvalues $\lambda =\left[\lambda_{1},\lambda_{2},\ldots ,\lambda_{N}\right]$. In this work, the vibration signal is constructed into the horizontal visibility graph (HVG) [20]. For a given a time series $\left\{x_{1},x_{2},\ldots ,x_{n}\right\}$, if $x_{i}$ and $x_{j}$ satisfy Equation (1) in the time series, $v_{i}$ and $v_{j}$ are connected vertices in the HVG.

(1) $$x_{i},x_{j}>x_{n},i<\forall n<j$$

**Figure 1:**
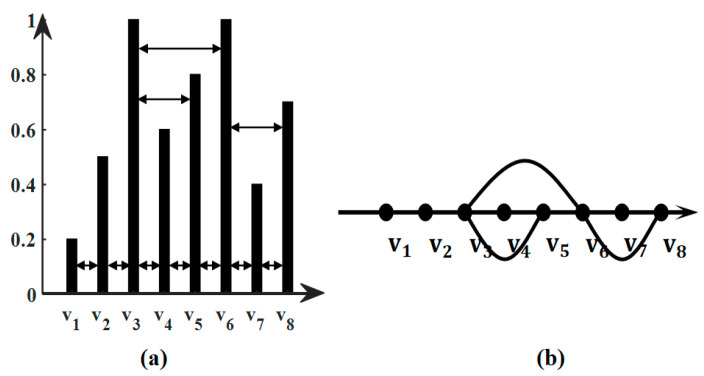
An example of a horizontal visibility graph: (**a**) time series data value; (**b**) horizontal visibility graph.

A time series and its HVG are shown in Figure 1. Figure 1a shows the bar plot of a time series [0.2 0.5 1.0 0.6 0.8 1.0 0.4 0.7]. Figure 1b is the HVG converted from the time series. Each data value in the time series corresponds to each vertex in the HVG.

It is worth noting that the weight value of edges $w_{ij}$ directly influences the results of graph signal processing methods. The main weighting methods are described below.

(1) The constant weighting method describes the connection of vertices but neglects the feature diversity of vertices. The weight value of edges $w_{ij}$ is defined as Equation (2)

(2) $$w_{ij}=1$$

(2) The Gaussian kernel weighting method utilizes the Gaussian function for calculating the weight value of edges $w_{ij}$, as shown in Equation (3).

(3) $$w_{ij}=\exp\left(-{\left\|x_{i}-x_{j}\right\|}^{2}/2\sigma^{2}\right)$$

where σ is the width of the Gaussian kernels.

(3) The Euclidean weighting method utilizes the Euclidean distance for calculating the weight value of edges $w_{ij}$, as shown in Equation (4).

(4) $$w_{ij}=\left\|x_{i}-x_{j}\right\|$$

(4) The cosine weighting method is defined as Equation (5).

(5) $$w_{ij}=1-\frac{\langle x_{i},x_{j}\rangle }{{\left|\left|x_{i}\right|\right|}_{2}{\left|\left|x_{j}\right|\right|}_{2}}$$

### 2.2. Graph Coarsening

**Figure 2:**
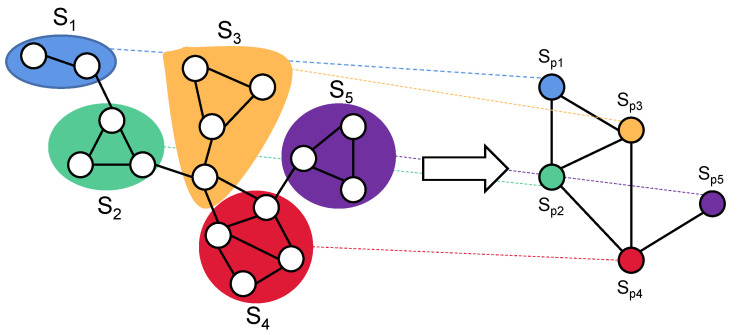
An example illustrating the graph coarsening procedure with five graph partitions.

Graph coarsening aims to generate a coarse-grained graph of reduced size while preserving the original graph properties. Let the coarse-grained graph $G_{c}=\left\{V_{c},E_{c},W_{c}\right\}$ with $n=\left|V_{c}\right|$ be obtained from the original graph G. For graph coarsening, a set of non-overlapping graph partitions $P=\{S_{1},S_{2},\ldots ,S_{n}\}\subset V$ is firstly selected and each partition $S_{p}$ corresponds to a super-node $s_{p}$. An example illustrating the graph coarsening process with five graph partitions is shown in Figure 2. Super-nodes ($s_{p},s_{q}$) are connected by super-edges $W_{c}(p,q)$ whose weight values are equal to the accumulative edge weights between nodes in graph partitions ($S_{p},S_{q}$), as shown in Equation (6).

(6) $$W_{c}\left(p,q\right)=w\left(S_{p},S_{q}\right)=\sum_{v_{i}\in S_{p},{v_{j}\in S}_{q}}W\left(i,j\right)$$

Suppose the partition matrix $P\in R^{n\times N}$ contains columns which are indicator vectors for dividing nodes of the original graph into graph partitions of the coarse-grained graph. P is defined as Equation (7).

(7) $$P\left(p,i\right)=\left\{\begin{array}{l} 1,\;\;\;\;\;\;\;if\;v_{i}\in S_{p}\\ 0,\;\;\;\;\;otherwise \end{array}\right.$$

According to Equation (6), the weighted adjacency matrix of the coarse-grained graph $W_{c}$ can be obtained and defined as Equation (8).

(8) $$W_{c}=PWP^{T}$$

In this work, the spectral graph coarsening (SGC) method is introduced to calculate the weighted adjacency matrix [21]. In SGC, an iteration loop is implemented to search for different possible combinations of eigenvectors and the k-means clustering algorithm is applied to select the graph coarsening option with the minimum cost. The k-means cost function is defined as Equation (9).

(9) $$F\left(U_{k1},P_{k1}^{*}\right)=\sum_{i=1}^{N}{\left(r\left(i\right)-\sum_{j\in S_{i}}\frac{r\left(j\right)}{S_{i}}\right)}^{2}\;$$

where $P_{k1}^{*}$ is graph partitions and $U_{k1}=\left[U\left(1:k_{1}\right);U\left(k_{2}+1:N\right)\right]$, in which $k_{1}$ is defined as Equation (10) and $k_{2}=N-n+k_{1}$ in the iteration.

(10) $$k_{1}=\left\{\begin{array}{c} n,\;\;\;\;\;\;\;\;\;\;\;\;\;\;\;\;\;\;\;\;\;\;\;\;\;\;\;\;\;\;\;\;\;\;\;\;\;\;\;\;\;\;\;\;\;\;\;\;\;\;\;\;\;\;\;\;\;\;\;\;\;\;\;\;\;\;\;\;\;\;\;\;\;\;\;\;\;\;\;\;\;\;\;\;\;\;\;\;\;\;\;\;\mathrm{if}\;\lambda \left(N\right)\leq 1\;\\ k_{1}={argmin}_{k}\left\{k:\lambda \left(k\right)\leq 1,k\leq n,\lambda \left(N-n+k+1\right)>1\right\},\;\mathrm{otherwise} \end{array}\right.$$

The partition matrix is obtained by minimizing the k-means cost in Equation (11).

(11) $$P^{*}=argminF\left(U_{k1},P_{k1}^{*}\right)$$

Substituting Equation (11) into Equation (8), the weighted adjacency matrix of the coarse-grained graph can be obtained.

### 2.3. Graph Permutation Entropy

Graph permutation entropy is a measurement of the regularity of graphs obtained by combining the vertices’ features with the topology of the graphs. There are four procedures for calculating graph permutation entropy ${PE}_{G}$, as follows:

(1) The embedding vector $y_{k}^{m,\tau }\in R^{m}$ is firstly constructed as Equation (12).

(12) $$Y_{k}^{m,\tau }={\left(y_{i}^{k\tau }\right)}_{k=0}^{m-1}=\left(y_{i}^{0},y_{i}^{\tau },\ldots ,y_{i}^{\left(m-1\right)\tau }\right),i=1,2,\ldots ,N$$

where m is the embedding dimension (2≤m∈N), τ is the delay time (τ∈N), and $y_{i}^{k\tau }$ is defined as Equation (13).

(13) $$y_{i}^{k\tau }=\frac{1}{\left|H_{k\tau }\left(i\right)\right|}\sum_{j\in H_{k\tau }\left(i\right)}x_{j}=\frac{1}{\left|H_{k\tau }\left(i\right)\right|}{\left(W^{k\tau }X\right)}_{i}$$

where $H_{k\tau }(i)$ is the set of all vertices connected to the vertex i with a walk on kτ edges. It follows that $y_{i}^{0}=x_{i}$ and $y_{i}^{1}=(I-\Delta )x_{i}$.

(2) The elements in embedding vector $y_{k}^{m,\tau }$ are associated with integer numbers from 1 to m and then rearranged in increasing order. Hence, there are m! permutations in which k distinct permutations exist (k≤m!).
(3) The probability of distinct permutations is denoted by $P_{r}$. According to Shannon entropy, the graph permutation entropy for the k distinct permutations can be defined as Equation (14).

(14) $${PE}_{G}\left(m,\tau \right)=-\sum_{r=1}^{R}P\left(r\right)lnP\left(r\right)$$

(4) When $P_{r}=1/m!\;$, the probability values of distinct permutations are the same, and hence ${PE}_{G}$ has the highest value $\ln\left(m!\right)$. Finally, the normalized procedure is implemented as shown in Equation (15).

(15) $${PE}_{G}\left(m,\tau \right)=\frac{{PE}_{G}\left(m,\tau \right)}{ln\left(m!\right)}$$

## 3. Graph Multi-Scale Permutation Entropy for Bearing Fault Diagnosis

In this work, graph multi-scale permutation entropy is proposed for bearing fault diagnosis. There are four main procedures in the proposed method, as follows:

(1) Graph transform. The vibration signal samples from bearings are transformed into HVGs.
(2) Graph coarsening with multiple scales. Multi-scale coarse-grained graphs with different numbers of vertices are obtained for each HVG using the SGC method.
(3) Graph permutation entropy calculation. The graph permutation entropy values of all multi-scale coarse-grained graphs are calculated for constructing feature vectors of each sample.
(4) Classifier training. Features are divided into training data and testing data. The support vector machine (SVM) is trained by feeding the training data and then the testing data are used to evaluate the classifier. Finally, the bearing fault classifier can be obtained.

## 4. Experiment

To verify the effectiveness of the graph multi-scale permutation entropy method, two cases, A and B, were conducted using open-source data and laboratory data, respectively.

### 4.1. Case A: Vibration Signals from the Public Dataset

Bearing data from the Bearing Data Center of Case Western Reserve University were used to select the parameters of MPE_G_ in this case. In this work, the bearing data with a damage diameter of 7 mils were selected as test signals. The motor speed was 1797 rpm and the load was 0 Hp. The sampling frequency was 12 kHz and there were 1024 sampling points for each sample. Each health status includes 20 samples; there are 80 samples for four health statuses, including rolling ball fault (RF), inner race fault (IF), outer race fault (OF), and healthy (H) bearing. For each health status, 5 samples were randomly chosen for training and the remaining 15 samples were used for testing; thus, a total of 20 training samples and 60 testing samples were obtained.

**Figure 3:**
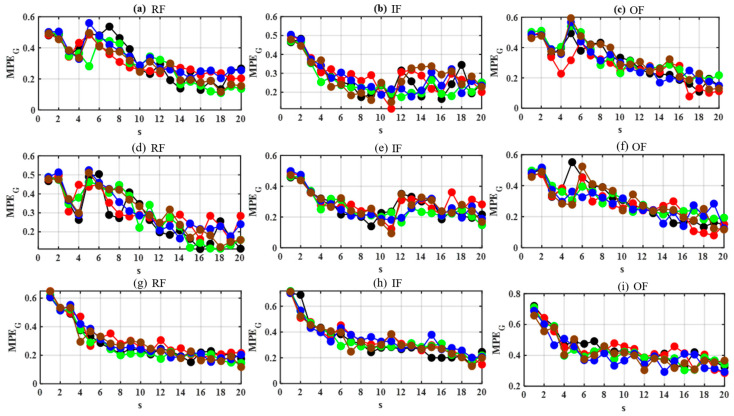
Graph multi-scale permutation entropy values using three different weighting methods. (**a**–**c**) using the constant weighting method; (**d**–**f**) using the Gaussian kernel weighting method; (**g**–**i**) using the Euclidean weighting method.

To test the influence of different weighting methods, a comparison experiment was conducted using three different weighting methods, i.e., the constant weighting method, the Gaussian kernel weighting method, and the Euclidean weighting method. Figure 3 shows the graph multi-scale permutation entropy (MPE_G_) values of samples with RF, IF, and OF using different weighting methods. For each health status, 5 samples were weighted using the three weighting methods to calculate MPEG and represented in different color lines. It can be seen from Figure 3 that MPE_G_ decreases with the increase in the scale factor s. For samples exhibiting equivalent health conditions, the utilization of Euclidean kernel weighting yields MPE_G_ values that converge more closely across varying scale factors. This phenomenon is indicative of reduced intra-class variability. The variances in MPE_G_ values using different weighting methods are calculated as shown in Table 1. Table 1 demonstrates that the variance in MPE_G_ under various health conditions is minimized when employing the Euclidean distance weighting method. It suggests superior performance characterized by enhanced discriminatory capacity and classification precision. Hence, the Euclidean kernel weighting method is used to calculate MPE_G_ in this work.

**Figure 4:**
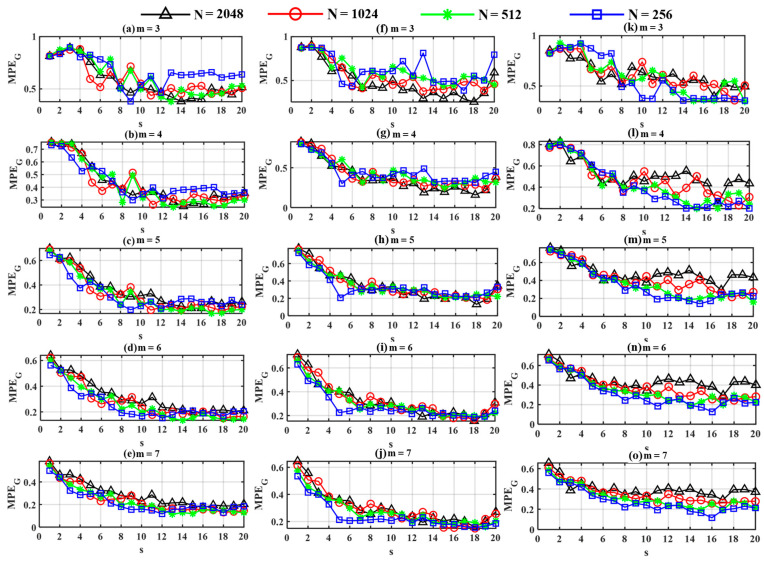
Graph multi-scale permutation entropy values with different *N* and *m*: (**a**–**e**) rolling ball fault; (**f**–**j**) inner race fault; (**k**–**o**) outer race fault.

The selection of parameters, such as embedding dimension m, sample length N, and time delay τ, directly influences the value of MPE_G_. A comparison experiment was conducted using samples with different N=256, 512, 1024, 2048 and m=3, 4, 5, 6, 7. In the experiment, s ranged from 1 to 20 and τ was set to 1, respectively. Figure 4 shows MPE_G_ with respect to s under different parameter settings. It can be seen from the figure that the MPE_G_ tends to decrease as the value of s increases. Also, some MPE_G_ values demonstrate substantial and irregular fluctuations when m is 3 or 4. In contrast, when m is 5, 6, or 7, there is a trend of gradually decreasing MPE_G_ values, which tends to stabilize after s reaches 10. Hence, MPE_G_ values with s = 1–20 were selected as features for classification in this work according to experience.

Furthermore, Figure 4 shows that the intra-class distances of MPE_G_ values for the same s tend to decrease with increasing values of m. However, a larger value of *m* leads to an increase in computation time. The CPU model utilized in this experiment was the Intel(R) Core(TM) i7-7700K, sourced from Intel Corporation, headquartered in Santa Clara, CA, USA, featuring 32 GB of RAM. Additionally, the graphics card employed was the GeForce GTX 1660 Super, manufactured by NVIDIA Corporation, based in Santa Clara, CA, USA, equipped with 6 GB of video memory. Table 2 illustrates the runtime for computing MPE_G_ values under different values of m when N is set to 1024. It is evident that when *m* is increased to 7, the computation time becomes three times that of *m* = 6, indicating a significant increase. Hence, *m* was set to 6 in this work, after taking all factors into consideration.

The value of *N* also significantly influences the computation time for calculating MPE_G_ values. Table 3 is the runtime for computing MPE_G_ values under different values of *N*. Table 3 shows the runtime increases with the increase in *N*. This effect is particularly pronounced when *N* is set to 2048, where the efficiency drops by approximately 85% compared to when *N* is set to 1024. However, selecting small values of *N* may lead to the omission of critical fault information due to insufficient data length. Hence, *N* was set to 1024 in this work.

**Figure 5:**
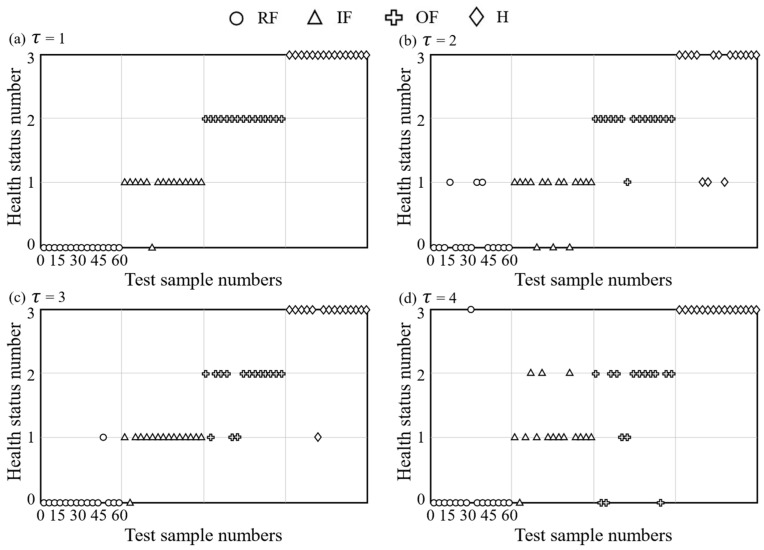
Classification results of bearing faults with different values of τ.

Another experiment was conducted to test the effect of testing time delay τ values on the classification results. In this experiment, four health statuses (RF, IF, OF, H) were assigned the numerical labels 0, 1, 2, and 3, respectively. For each health status, five samples were randomly chosen as training samples, while fifteen samples were designated as testing samples. The sequence of the 60 testing samples is as follows: RF (1–15), IF (16–30), OF (31–45), and H (46–60). Figure 5 is the classification results of bearing faults with different values of τ. It can be seen from Figure 5 that the classification accuracy for τ values of 1, 2, 3, and 4 is 98.3%, 83.3%, 90%, and 83.3% respectively. The highest accuracy was achieved when τ was set to 1. Therefore, τ was selected as 1 in this work.

**Figure 6:**
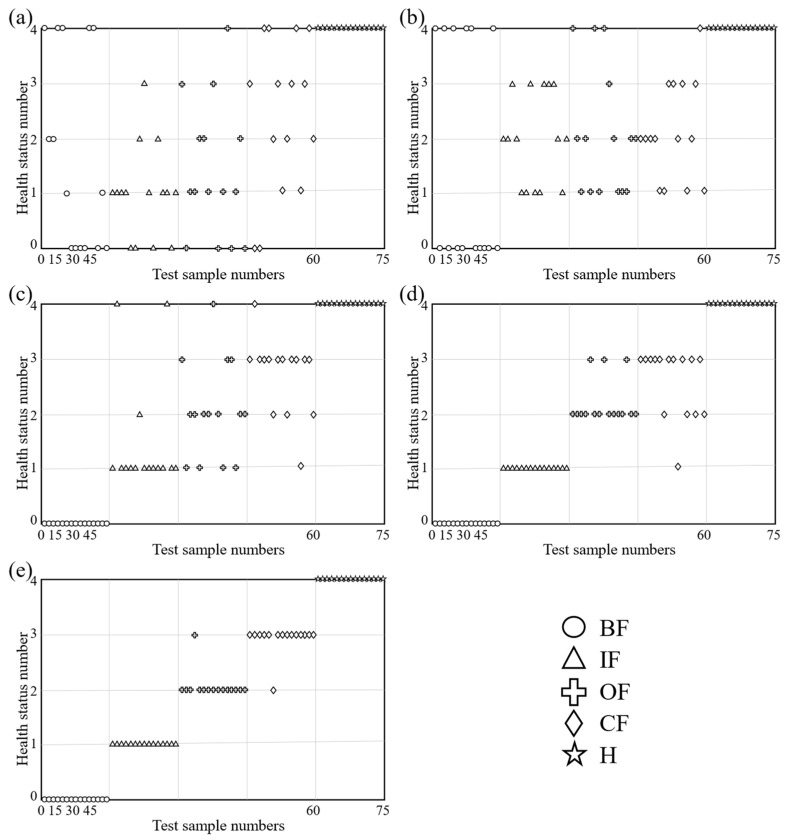
Classification results using five different entropies in Case A. (**a**) PE, (**b**) PE_G_, (**c**) MPE, (**d**) MAE, (**e**) MPE_G_.

A comparison experiment was implemented to test the robustness of MPE_G_. Bearing data from South Ural State University [22] were used in this experiment. In this work, the linear acceleration in the bearing data was selected as the test signal. The motor speed was 1200 rpm and the sampling frequency was 31.175 kHz. Considering that there are 1024 sampling points for each sample, the bearing data were downsampled to 10 kHz to ensure that each sample contained information from two revolutions of the test bearing. Each health status includes 20 samples; there were thus 100 samples for five health statuses, including rolling ball fault (BF), inner race fault (IF), outer race fault (OF), combination fault (CF), and healthy (H) bearing. For each health status, 5 samples were randomly chosen for training and the remaining 15 samples were used for testing; thus, a total of 25 training samples and 75 testing samples were obtained. The other four entropies, i.e., permutation entropy (PE) [23], graph permutation entropy (PE_G_) [19], multi-scale permutation entropy (MPE) [24], and multi-scale approximate entropy (MAE) [25], were added to the experiment. Figure 6 shows the classification results when using five different entropies, in which different colors represent different operating conditions. It can be seen from Figure 6 that the classification accuracies for the four methods, PE, PE_G_, MPE, and MAE, are 48%, 50.7%, 78.7%, and 89.3%, respectively. The MPE_G_ method has only two misclassified samples, achieving an accuracy of 97.3%, which is 49.3%, 46.6%, 18.6%, and 8% higher than the aforementioned four methods, respectively. These results demonstrate that the proposed method is superior to conventional methods for identifying different kinds of bearing faults.

### 4.2. Case B: Vibration Signals from the Laboratory

**Figure 7:**
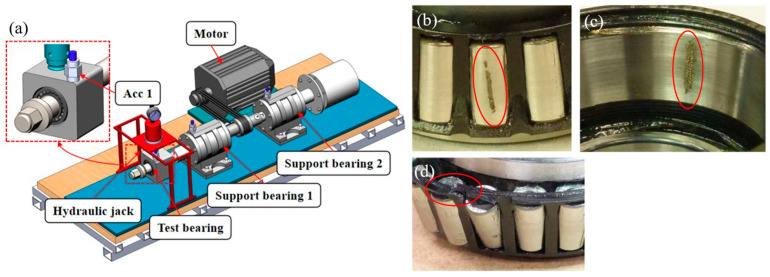
The bearing test rig and faulty bearings in in our laboratory: (**a**) test rig, (**b**) roller fault, (**c**) outer race fault, (**d**) cage fault.

Vibration signals were collected from the test rig as shown in Figure 7a, undertaken in our laboratory. The test faulty bearings are shown in Figure 7b. In this experiment, an MMF Type KS76-100/1 instrumentation-standard piezoelectric accelerometer (Acc 1) was used to measure vibration and was mounted on the test bearing by means of a strong magnet. The accelerometer was calibrated with an MMF Type VC20 vibration calibrator. The parameters of the bearing are shown in Table 4. Data were collected under four kinds of health status, i.e., roller fault (RF), outer race fault (OF), cage fault (CF), and healthy (H), as described in Table 5. Data for each health status were collected at three different rotational speeds, with 7 samples collected at each speed, resulting in a total of 21 samples per health status. During the experiment, 6 samples were chosen from each health status dataset as the training set, while the remaining 15 samples were allocated as the testing set. In total, there were 24 training samples and 60 testing samples.

To further test the robustness and advantages of the proposed method, the rotational speeds were set to 300 rpm, 400 rpm, and 500 rpm. As mentioned in Case A, the point number *N* of each sample was 1024. In this case, each sample contains information from two revolutions of the test bearing. Hence, the sampling frequency was set to 2 kHz, 2.5 kHz, and 3 kHz, respectively. Also, a hydraulic jack was used to apply a constant vertical load of 800 N over the test bearing.

**Figure 8:**
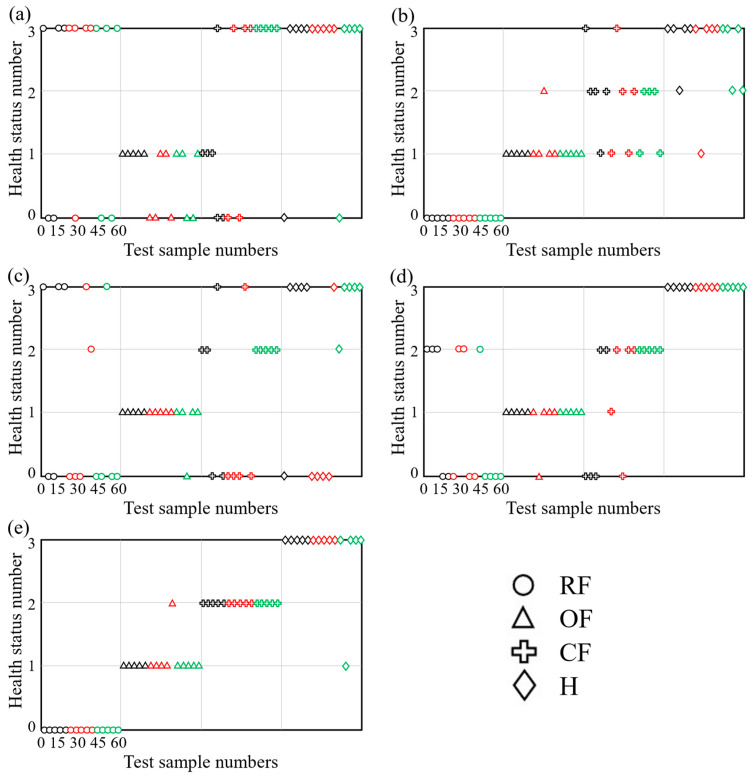
Classification results using five different entropies in Case 2: (**a**) PE, (**b**) PE_G_, (**c**) MPE, (**d**) MAE, (**e**) MPE_G_.

The four health statuses (RF, OF, CF, H) are labeled as 0, 1, 2, and 3, respectively. The sequence of the 60 testing samples is as follows: RF (1–15), OF (16–30), CF (31–45), and H (46–60). The other four entropies mentioned in Case 1 were used for comparison against the proposed method in this section as well. The comparison results obtained are illustrated in Figure 8. In this figure, black represents a sample with a rotational speed of 300 rpm, red represents a speed of 400 rpm, and green corresponds to a speed of 500 rpm. It can be seen from Figure 8 that the classification accuracies for the four methods, PE, PE_G_, MPE, and MAE, are 46.7%, 80%, 65%, 80%. The MPE_G_ method has only one misclassified sample, achieving an accuracy of 96.7%, which is 50%, 16.7%, 31.7%, and 16.7% higher than the aforementioned four methods, respectively. The results further validate the proposed method’s accuracy and robustness when dealing with data at different rotational speeds.

## 5. Conclusions

A novel graph multi-scale permutation entropy (MPE_G_) method is proposed for bearing fault diagnosis in this work. In the proposed method, vibration signals are first transformed into horizontal visibility graphs. Secondly, the graphs are coarsened at multiple scales to obtain multiple coarse-grained graphs. Thirdly, the permutation entropy values of these coarse-grained graphs are then employed as a multi-dimensional feature input for training a support vector machine (SVM). Comparison experiments were implemented using two different datasets to test the proposed method’s performance. Based on the experimental results, the following conclusions can be drawn:

1. The results of the comparison experiments in Case A demonstrate that the MPE_G_ method is superior to conventional methods (PE, PEG, MPE, and MAE) for identifying different kinds of bearing faults.
2. The proposed method demonstrates strong robustness when dealing with data from various operating conditions. It outperforms traditional methods (PE, PE_G_, MPE, and MAE) in fault diagnosis. In Case B, the accuracy of the proposed method is 16% to 50% higher when compared to other methods.
3. This work validates the effectiveness and advantages of applying the MPE_G_ method to rolling bearing fault diagnosis. However, it has not yet been applied to diagnose other equipment faults in machinery. Therefore, the efficacy of the MPE_G_ method in other fault diagnostics needs further exploration.

## Figures and Tables

**Table 1:** Variance among MPE_G_ values using different weighting methods.

	Constant Weighting	Gaussian Kernel Weighting	Euclidean Weighting
RF	0.01534	0.01111	0.00951
IF	0.01253	0.01050	0.00871
OF	0.01820	0.01295	0.01152

**Table 2:** Runtime for computing MPE_G_ values under different values of *m*.

*m*	3	4	5	6	7
Time	0.07 s	0.1 s	0.18 s	0.55 s	2.9 s

**Table 3:** Runtime for computing MPE_G_ values under different values of *N*.

*N*	256	512	1024	2048
Time	2 min	8 min	40 min	9 h

**Table 4:** Test bearing parameters.

Type	Bearing No.	Number of Rollers per Cage	Roller Diameter	Pitch Diameter	Roller Angle
Parameters	TAROL 130/230-U-TVP	22	24 mm	187 mm	6.9°

**Table 5:** Health status of test bearings.

Bearing ID	Description
RF	A minor scratch along the roller, inflicted by an electrical discharge engraver
OF	A minor scratch across the race (axial direction), inflicted by a combination of an engraver and small grinder
CF	The bearing cage cracked in one place, achieved by cutting and applying excess force with a screwdriver
H	Healthy bearing

## Data Availability

The data cannot be made publicly available upon publication because they are owned by a third party and the terms of use prevent public distribution. The data that support the findings of this study are available upon reasonable request from the authors.
